# Mechanistic modeling of the SARS-CoV-2 disease map

**DOI:** 10.1186/s13040-021-00234-1

**Published:** 2021-01-21

**Authors:** Kinza Rian, Marina Esteban-Medina, Marta R. Hidalgo, Cankut Çubuk, Matias M. Falco, Carlos Loucera, Devrim Gunyel, Marek Ostaszewski, María Peña-Chilet, Joaquín Dopazo

**Affiliations:** 1 Bioinformatics Area, Fundación Progreso y Salud (FPS), Hospital Virgen del Rocío, Sevilla, Spain; 2 Computational Systems Medicine, Institute of Biomedicine of Seville (IBIS), Hospital Virgen del Rocio, 41013 Sevilla, Spain; 3 Bioinformatics and Biostatistics Unit, Centro de Investigación Príncipe Felipe (CIPF), 46012 Valencia, Spain; 4 Bioinformatics in RareDiseases (BiER), Centro de Investigación Biomédica en Red de Enfermedades Raras (CIBERER), Sevilla, Spain; 5 Luxembourg Centre for Systems Biomedicine, University of Luxembourg, L-4367 Belvaux, Luxembourg; 6 Functional Genomics Node (INB-ELIXIR-es), Sevilla, Spain

**Keywords:** Mechanistic modeling, Signaling pathway, Drug discovery, COVID-19, Systems biology

## Abstract

Here we present a web interface that implements a comprehensive mechanistic model of the SARS-CoV-2 disease map. In this framework, the detailed activity of the human signaling circuits related to the viral infection, covering from the entry and replication mechanisms to the downstream consequences as inflammation and antigenic response, can be inferred from gene expression experiments. Moreover, the effect of potential interventions, such as knock-downs, or drug effects (currently the system models the effect of more than 8000 DrugBank drugs) can be studied. This freely available tool not only provides an unprecedentedly detailed view of the mechanisms of viral invasion and the consequences in the cell but has also the potential of becoming an invaluable asset in the search for efficient antiviral treatments.

## Introduction

The recent pandemic of COVID-19 (Coronavirus Disease-2019), an emerging respiratory disease caused by the SARS-CoV-2 virus, which spread more efficiently than previous highly pathogenic coronaviruses SARS-CoV and MERS-CoV, has led to a tremendous toll of affected cases and over 500,000 fatalities in more than 200 countries since its first outbreak in late 2019 [1]. Precisely due to the rapid transmission of this novel pathogen, no antiviral drugs or vaccines are available for SARS-CoV-2.

Understanding the molecular mechanisms that mediate SARS-CoV-2 infection is key for the rapid development of efficient preventive or therapeutic interventions against the COVID-19. A comprehensive description of such molecular mechanisms is represented in the corresponding disease map, that is, the sub-module of the whole pathway of known human protein functional interactions that summarize details of the disease mechanism and consequently are relevant for understanding the disease [2]. The recent availability of a detailed catalog of viral-human protein interactions [3] has facilitated the construction of a first version of a map of the human molecular pathways involved in the viral infection and downstream consequences [4].

Disease maps are repositories of knowledge of disease-relevant mechanisms that provide qualitative guidance for the interpretation of experimental findings [2]. Actually, disease maps are the supporting foundation of different tools able to model the information contained in them in order to provide a detailed quantitative explanation for experimental results [5]. In particular, mechanistic models of disease maps are becoming increasingly relevant for genomic data interpretation because they provide a natural link between omics data measurements and cell behavior and outcome [6], which ultimately accounts for the phenotype of the infection. The knowledge of these links allows a better understanding of the molecular mechanisms of the viral infection and the responses to drugs. Actually, mechanistic models of human signaling [7] or metabolic pathways [8] have been successfully used to uncover specific molecular mechanisms behind different cancers [7, 9–11], rare [12] and common [13] diseases, to reveal mechanisms of action of drugs [14], and dissecting them at single cell level [15], to suggest personalized treatments [16, 17] and in other biologically interesting scenarios [18, 19]. Basically, mechanistic models analyze experimental values in the context of the disease map information, which is used to point out the relevant aspects of the molecular mechanisms behind the experiment. It is important to note that this assessment is made from a systems biology perspective, in the holistic context of the disease map, and considers the functional interactions among the gene products as described in the map. Typically, these experimental values are gene expression transcriptomic data, although other data such as proteomic, phosphoproteomic, genomic [20], or even methylomics, can also be used. Interestingly, beyond its usefulness for the functional interpretation of experimental results, the most remarkable property of mechanistic models is that they can be used to predict the effects of interventions (inhibitions, over-activations, drugs, etc., alone or in combinations) over proteins of the map in the condition studied [21]. Therefore, this opens the possibility of using these models for exploring new therapeutic options as well [22].

## Methods

To construct a first approach to the COVID-19 disease map, the SARS-CoV-2 virus-human interactome was firstly expanded from existing KEGG pathways [23] to define regions within the whole set KEGG pathways that potentially account for the molecular mechanism of the viral infection and the downstream consequences. Pathways are composed of individual signaling circuits (sub-pathway that describes the chain of signal transduction that connects a receptor protein to an effector protein) whose functionalities can be described by the UniProt [24] functional annotations of their effector nodes [7, 25]. It order to restrict the map to those circuits relevant for the COVID-19 disease mechanism, only signaling circuits with at least one UniProt [24] function that fit in one of these virus-related categories: 1) Host-virus interaction, 2) inflammatory response, 3) immune activity, 4) antiviral defense, 5) endocytosis were selected to define the COVID-19 disease map. The model presented here is a part of an ongoing more detailed repository of SARS-CoV-2 mechanisms, the COVID-19 Disease Map, in construction by an international community, whose most recent version is available at: 10.17881/covid19-disease-map. In addition to the human version of the COVID-19 map, versions for animal models, like mouse or rat, using the homologous pathways are also provided by the tool [4].

The mechanistic model implemented here takes a directed graph (in this case a first version of the COVID-19 map, and in the future new versions as these are released), and extracts from it the collection of signaling circuits that connect receptor nodes to effector nodes. The signal transduction across such circuits, S_n_, is estimated using gene expression values as proxies of protein activity [26] using the following recursive equation [7]:

1 $$ {S}_n={\upsilon}_n\bullet \left(1-\prod \limits_{s_a\in A}\left(1-{s}_a\right)\right)\cdotp \prod \limits_{s_i\in I}\left(1-{s}_i\right) $$

*S*_*n*_ is the signal intensity for the current node *n*, *v*_*n*_ is its normalized gene expression value, *A* is the set of activation signals (*s*_*a*_), arriving to the current node from activation edges, *I*is the set of inhibitory signals (*s*_*i*_) arriving to the node from inhibition edges. The *S*_*n*_ values of circuits are further used in comparisons to detect increases or decreases in signaling activity (and consequently in the corresponding cell functionality), or to infer the effect of interventions in signaling or the potential resulting phenotype of mutations.

### Implementation

**Fig. 1:**
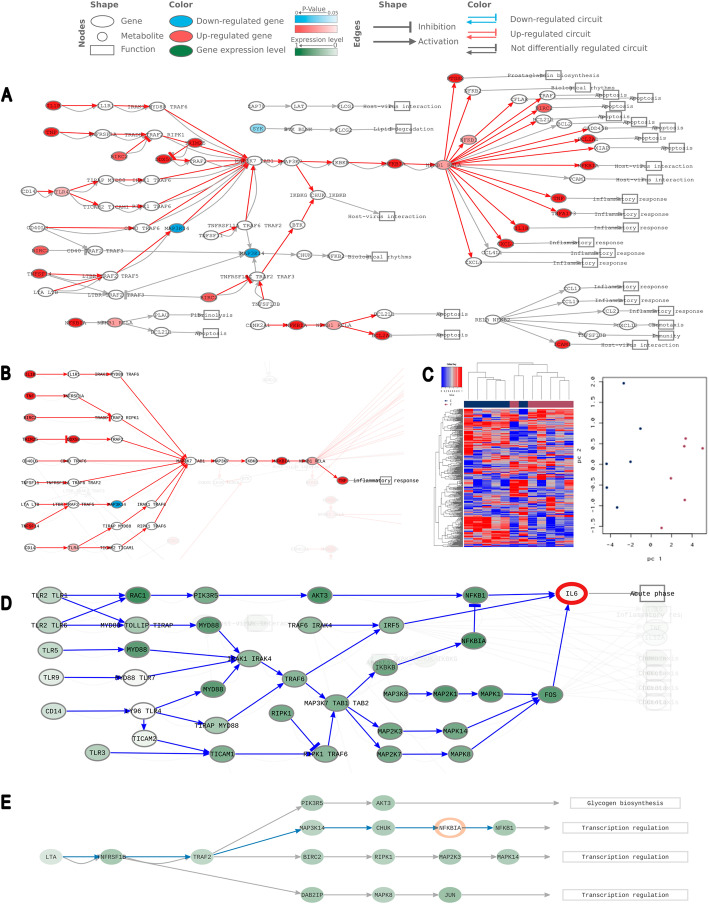
**a** Activation pattern of NF-KB pathway in lung cell lines infected with SARS-CoV-2. **b** Detail of NF-KB pathway’s circuit with TNF as effector protein. **c** Heatmap representing activation values of all the circuits in COVID-19 disease map (left) and a representation of a Principal Component Analysis based on signaling profiles of the samples that clearly segregates the two conditions studied: controls are represented in dark blue and cases in purple (right). **d** An example of the *Perturbation effect* option simulating the effect of Siltuximab (targeting *IL6* protein*)* in Toll-like signaling pathway. Individual gene expression levels in each node are represented green scale. **e** An example of the *Variant interpreter* option simulating the effect of a loss of function mutation in *NFKBIA* protein over lung tissue pathway activity in the TNF signaling pathway. Mean values of gene expression levels in each node are represented in green scale

Here, we present the first implementation of a mechanistic model of the SARS-CoV-2 infection in a user-friendly web interface. The model used here implements the *HiPathia* [7] algorithm, which has demonstrated to outperform other competing algorithms in a recent benchmarking [25]. The mechanistic model implemented in *HiPathia* has been successfully used to understand the disease mechanisms behind different cancers [7] and was able to predict cancer vulnerabilities with a high precision [9]. The model has been implemented in a user-friendly web application that inputs normalized gene expression values (or similar proteomics or phosphoproteomic values) and can be found at http://hipathia.babelomics.org/covid19/. As an example, we carried out some analyses that involve a case-control differential signaling analysis using a recently published gene expression experiment [27] with human lung cell lines infected with SARS-CoV-2 (GEO id: GSE147507, the dataset GSE147507_RawReadCounts_Human.ts). The infected cells showed a differential activation pattern in circuits related to virus entrance to cell, activation of immune, inflammatory and other virus-triggered responses (see Fig. 1a and Table 1 for a detailed list of differentially activated signaling circuits and Table 2 for detail on the differentially activated cell functionalities). Interestingly, several of the deregulated pathways include *TNF*, a target gene of chloroquine, one of the drugs with promising results against COVID-19 [28]. Moreover, NF-kB signaling pathway has been highlighted in several studies as one of the main pathways responsible for COVID-19 progression [29] (Fig. 1 B). Figure 1c depicts the heathmap of signaling activity profiles that discriminate the two classes of samples (cases and controls) compared. The results found are consistent with those of previous analyses with the same data, where a modest but generalized response of mechanisms related to immune response and inflammatory processes, such as response to chemokine and cytokines, virus defense-related processes, and other general functions of cell like apoptosis or cell differentiation was demonstrated [27]. Interestingly, the processes highlighted by the authors are response to chemokines and interferon-related signaling pathways. Both processes are highly associated with pathways in whose circuits our model detect significant deregulations (see Table 1), such as Toll-like and TNF signaling pathways. The relation between chemokines, interferon and Toll-like signaling is well-known [30, 31], moreover, interferon is a key player in TNF signaling pathway [32], and both signaling pathways are interrelated. Beyong the pure interpretation of the results, another novel and very useful option of the implementation of the model is the *Perturbation effect*. It allows estimating the effect of interventions (inhibitions or overexpression) across the signaling circuits of the model in a given condition. Moreover, the effect of more than 8000 targeted drugs from DrugBank can be predicted by selecting them, individually or in combinations. Figure 1d shows and example of the *Perturbation effect* option in which the impact of Siltuximab, a drug in study in patients with COVID-19 respiratory failure [33] which targets protein *IL6*, is simulated. Individual gene expression levels in each node are represented in shades of grayish green. Figure 1e, shows an example of the *Variant interpreter* option simulating the effect of a loss of function mutation in *NFKBIA* gene product over lung tissue pathway activity. One of the affected circuits in TNF signaling pathway is shown. Mean values of gene expression levels in each node are represented in shades of grayish green. A detailed description of the usage of the tool can be found in the accompanying help and tutorial.

**Table 1:** Circuits from CoV-Hipathia differentially activated in lung cell lines infected with SARS-CoV-2

KEGG pathway: effector gene/s	UP/ DOWN	statistic	***p***-value^**a**^	FDR^**b**^	FC^**c**^	log FC
MAPK signaling pathway: NLK	UP	2.882	2.16E-03	0.023	1.098	0.135
MAPK signaling pathway: STK3	UP	2.882	2.16E-03	0.023	1.209	0.274
MAPK signaling pathway: ECSIT, TRAF6	UP	2.882	2.16E-03	0.023	1.182	0.241
Ras signaling pathway: REL	UP	2.882	2.16E-03	0.023	1.074	0.103
Ras signaling pathway: MLLT4	UP	2.882	2.16E-03	0.023	1.049	0.069
NF-kappa B signaling pathway: CFLAR	UP	2.882	2.16E-03	0.023	1.212	0.278
NF-kappa B signaling pathway: BIRC2	UP	2.882	2.16E-03	0.023	1.261	0.335
NF-kappa B signaling pathway: XIAP	UP	2.882	2.16E-03	0.023	1.176	0.234
NF-kappa B signaling pathway: BCL2L1	UP	2.882	2.16E-03	0.023	1.193	0.254
NF-kappa B signaling pathway: GADD45B	UP	2.882	2.16E-03	0.023	1.179	0.238
NF-kappa B signaling pathway: BCL2A1	UP	2.882	2.16E-03	0.023	1.570	0.651
NF-kappa B signaling pathway: NFKB2	UP	2.882	2.16E-03	0.023	1.225	0.293
NF-kappa B signaling pathway: CXCL8	UP	2.882	2.16E-03	0.023	1.185	0.245
NF-kappa B signaling pathway: IL1B	UP	2.882	2.16E-03	0.023	1.341	0.424
NF-kappa B signaling pathway: TNFAIP3	UP	2.882	2.16E-03	0.023	1.311	0.390
NF-kappa B signaling pathway: NFKBIA	UP	2.882	2.16E-03	0.023	1.254	0.327
NF-kappa B signaling pathway: PTGS2	UP	2.882	2.16E-03	0.023	1.267	0.341
NF-kappa B signaling pathway: CXCL2	UP	2.882	2.16E-03	0.023	1.335	0.417
NF-kappa B signaling pathway: IKBKG, CHUK, IKBKB	UP	2.882	2.16E-03	0.023	1.110	0.151
NF-kappa B signaling pathway: BCL2L1	UP	2.882	2.16E-03	0.023	1.098	0.135
NF-kappa B signaling pathway: BCL2A1	UP	2.882	2.16E-03	0.023	1.448	0.534
HIF-1 signaling pathway: TIMP1	UP	2.882	2.16E-03	0.023	1.051	0.072
HIF-1 signaling pathway: TIMP1	UP	2.882	2.16E-03	0.023	1.051	0.072
HIF-1 signaling pathway: EDN1	UP	2.882	2.16E-03	0.023	1.121	0.164
HIF-1 signaling pathway: NOS2	UP	2.882	2.16E-03	0.023	1.585	0.664
HIF-1 signaling pathway: PDK1	UP	2.882	2.16E-03	0.023	1.037	0.052
HIF-1 signaling pathway: PGK1	UP	2.882	2.16E-03	0.023	1.048	0.068
HIF-1 signaling pathway: LDHA	UP	2.882	2.16E-03	0.023	1.047	0.066
mTOR signaling pathway: VEGFA	UP	2.882	2.16E-03	0.023	1.059	0.083
mTOR signaling pathway: TSC1	DOWN	−2.882	2.16E-03	0.023	0.946	−0.080
PI3K-Akt signaling pathway: BCL2L11	DOWN	−2.882	2.16E-03	0.023	0.918	−0.123
Apoptosis: FADD, TRADD	UP	2.882	2.16E-03	0.023	1.118	0.161
Apoptosis: IRAK3, MYD88	UP	2.882	2.16E-03	0.023	1.167	0.223
Toll-like receptor signaling pathway: CXCL10	UP	2.882	2.16E-03	0.023	1.518	0.602
Toll-like receptor signaling pathway: IFNA1	UP	2.882	2.16E-03	0.023	1.486	0.572
Toll-like receptor signaling pathway: IL1B	UP	2.882	2.16E-03	0.023	1.172	0.229
Toll-like receptor signaling pathway: IL6	UP	2.882	2.16E-03	0.023	1.526	0.610
RIG-I-like receptor signaling pathway: MAPK14	UP	2.882	2.16E-03	0.023	1.214	0.280
RIG-I-like receptor signaling pathway: MAVS, TMEM173	UP	2.882	2.16E-03	0.023	1.204	0.268
RIG-I-like receptor signaling pathway: IRF7	UP	2.882	2.16E-03	0.023	1.540	0.623
Ras signaling pathway: MAPK8	UP	2.722	4.33E-03	0.035	1.043	0.061
NF-kappa B signaling pathway: TRAF1	UP	2.722	4.33E-03	0.035	1.193	0.254
NF-kappa B signaling pathway: TNF	UP	2.722	4.33E-03	0.035	1.639	0.713
HIF-1 signaling pathway: TFRC	UP	2.722	4.33E-03	0.035	1.049	0.068
Sphingolipid signaling pathway: SMPD2	UP	2.722	4.33E-03	0.035	1.388	0.473
Apoptosis: FADD, TRADD	UP	2.722	4.33E-03	0.035	1.358	0.442
Apoptosis: TRAF2, RIPK1, TRADD	UP	2.722	4.33E-03	0.035	1.358	0.442
Toll-like receptor signaling pathway: TNF	UP	2.722	4.33E-03	0.035	1.434	0.520
TNF signaling pathway: CASP7	UP	2.722	4.33E-03	0.035	1.388	0.473
TNF signaling pathway: CASP3	UP	2.722	4.33E-03	0.035	1.371	0.455
TNF signaling pathway: BAG4	UP	2.722	4.33E-03	0.035	1.391	0.476

^a^ P-value is calculated using Wilcoxon test

^b^ FDR refers to p-value adjusted for multiple comparisons using Benjamini and Hochberg method

^c^ FC means Fold Change

**Table 2:** Functions from CoV-Hipathia differentially activated in lung cell lines infected with SARS-CoV-2

UniProt function	UP/DOWN	statistic	p-value^**a**^	FDR^**b**^	FC^**c**^	logFC
Innate immunity	UP	2.882	2.16E-03	0.018	1.048	0.067
Immunity	UP	2.882	2.16E-03	0.018	1.019	0.028
Inflammatory response	UP	2.882	2.16E-03	0.018	1.028	0.041
Antiviral defense	UP	2.882	2.16E-03	0.018	1.192	0.253
Pyrogen	UP	2.882	2.16E-03	0.018	1.177	0.235
Prostaglandin biosynthesis	UP	2.882	2.16E-03	0.018	1.267	0.341
Prostaglandin metabolism	UP	2.882	2.16E-03	0.018	1.267	0.341
Fatty acid biosynthesis	UP	2.882	2.16E-03	0.018	1.266	0.341
Lipid biosynthesis	UP	2.882	2.16E-03	0.018	1.266	0.341
Acute phase	UP	2.882	2.16E-03	0.018	1.526	0.610
Osteogenesis	UP	2.882	2.16E-03	0.018	1.181	0.241
Autophagy	DOWN	−2.722	4.33E-03	0.028	0.976	−0.035
Translation regulation	UP	2.722	4.33E-03	0.028	1.015	0.022
Sphingolipid metabolism	UP	2.722	4.33E-03	0.028	1.388	0.473
Necrosis	UP	2.562	8.66E-03	0.046	1.009	0.013
Fibrinolysis	UP	2.562	8.66E-03	0.046	1.103	0.141
Plasminogen activation	UP	2.562	8.66E-03	0.046	1.072	0.101

^a^ P-value is calculated using Wilcoxon test

^b^ FDR refers to p-value adjusted for multiple comparisons using Benjamini and Hochberg method

^c^ FC means Fold Change

Despite the limitations due to the few samples available, the results of the example clearly show the usefulness of this tool for modelling the repertoire of cell responses triggered by SARS-CoV-2, and the enormous potential that it has for future COVID-19 research and discovery of therapeutic interventions. Moreover, in spite of its short life CoV-Hipathia has already been quoted among other useful web tools to fight the COVID-19 pandemic [34] .

## Data Availability

The dataset used during the current study is available in the GEO database, [https://www.ncbi.nlm.nih.gov/geo/query/acc.cgi?acc=GSE147507], the COVID-19 Disease Map used here is available [10.5281/zenodo.3935733], and new versions will be made publicly available here [10.17881/covid19-disease-map]. This tool freely available at: http://hipathia.babelomics.org/covid19/
